# Targeted mesenchymal stem cell therapy equipped with a cell-tissue nanomatchmaker attenuates osteoarthritis progression

**DOI:** 10.1038/s41598-022-07969-9

**Published:** 2022-03-07

**Authors:** Nahid Nasiri, Samaneh Hosseini, Fakhreddin Reihani-Sabet, Mohamadreza Baghaban Eslaminejad

**Affiliations:** 1 Department of Stem Cells and Developmental Biology, Cell Science Research Center, Royan Institute for Stem Cell Biology and Technology, ACECR, Tehran, Iran; 2 Department of Embryology, Reproductive Biomedicine Research Center, Royan Institute for Reproductive Biomedicine, ACECR, Tehran, Iran; 3 Department of Cell Engineering, Cell Science Research Center, Royan Institute for Stem Cell Biology and Technology, ACECR, Tehran, Iran; 4 Department of Genetics, Reproductive Biomedicine Research Center, Royan Institute for Reproductive Biomedicine, ACECR, Tehran, Iran

**Keywords:** Adult stem cells, Mesenchymal stem cells, Regeneration

## Abstract

Mesenchymal stem cells (MSCs) are at the forefront of research for a wide range of diseases, including osteoarthritis (OA). Despite having attracted the attention of orthopedists, current MSC therapy techniques are limited by poor MSC implantation in tissue defects and lack of lateral tissue integration, which has restricted the efficacy of cell therapy to alleviate OA symptoms only. Here, we developed targeted MSC therapy for OA cartilage using a cell-tissue matchmaking nanoconstruct (C-TMN). C-TMN, as an MSC vehicle, consists of a central iron oxide nanoparticle armed with two types of antibodies, one directed at the MSC surface and the other against articular cartilage. We treated rat OA articular cartilage with intra-articular injections of C-TMN with and without exogenous MSCs. We observed substantial improvements in both symptomatic and radiographic OA caused by C-TMN, which was independent of exogenous MSCs. This new approach could predict a promising future for OA management.

## Introduction

Tissue engineering and cell therapy are two recent therapeutic possibilities for the management of diseases that cause progressive tissue damage, such as osteoarthritis (OA). OA is a rapidly growing health problem and a common cause of disability that affects up to 240 million middle-aged and elderly people worldwide^1^. Thus far, compensation of deep chondrocyte loss and hypocellularity in cartilage texture followed by OA progression has not occurred through conventional therapeutic options, including pain medications and classical surgical intervention, which operate the poor intrinsic regenerative responses of hyaline cartilage. Autologous chondrocyte implantation (ACI) and mesenchymal stem cell (MSC) therapy, as extrinsic cartilage repair factors, are two available advanced cell therapy techniques that have not been able to restore normal articular function in OA patients^2^. This is mainly due to the inadequate delivery of a sufficient number of therapeutic cells, whether recruited from bone marrow (BM) and vasculature or exogenously injected, to the injured articular cartilage (AC)^3, 4^. Moreover, a lack of lateral tissue integrity between newly formed repair cartilage and surrounding original tissue restricts the functionality of any regenerated tissue^5^.

There are various techniques in use that deliver therapeutic cells/drugs to osteoarthritic AC (OAC). Among these, cell surface modification or targeting by nanoparticles (NPs) are two main strategies for efficient cell delivery^6^. Modification of the therapeutic cell surface is accomplished using a method called “cell painting” in which an antibody (Ab) against a specific cartilage matrix antigen intercalates into the prechondrocyte cell membrane and enables the cell to bind to the cartilage^7^. Cell targeting peptides (CTPs) are another tool that targets OACs using cell surface changes with high degrees of specificity. Pi et al. explored a peptide sequence with strong chondrocyte binding ability^8^. Hu et al. synthetized an amino acid cartilage-binding peptide sequence with the dual binding ability to collagen type II (COL2) to a small construct composed of a drug carrier conjugated with a drug (a protease inhibitor). Their identified peptide caused a significant increase in drug retention time and inhibition of cartilage-degrading enzymes^9^. Despite the comparative therapeutic potential of MSC surface engineering methods, the need to change the cell membrane properties and associated safety concerns have limited their clinical applications. For example, covalent conjugation of peptides or Abs to the surface of therapeutic cells may affect membrane protein function and disturb relevant signaling patterns, leading to abnormal ligand-receptor binding and altered cell fate^10^.

As a safe alternative, NPs have a history of successful application in the clinic, including imaging, gene therapy and drug delivery. NPs exhibit unique flexibility for wide surface modifications that allow them to be used in various applications. Based on this capability, NPs can be attached to specific homing ligands and make appropriate ligand-receptor binding at the target site^11^. Moreover, NP-mediated cell-tissue attachment can create a three-dimensional (3D) microenvironment around the trapped cell, enabling efficient interaction with ECM, growth factors and other factors involved in tissue integration and cell differentiation^11, 12^.

Here, we have used a nanobiotechnology approach for targeted cell delivery into OACs by incorporating iron NPs equipped with antibodies. We conjugated two types of antibodies (Abs), one against CD90 on MSCs and the other for binding to COL2 on the AC surface, with a central iron NP core to produce a cell-tissue matchmaking nanoconstruct (C-TMN) for targeted delivery of MSCs to OACs. The Abs present at the surface of the iron core could link both exogenously and endogenously recruited CD90^+^ MSCs (and other CD90^+^ mesenchymal progenitor cells presented by different articular tissues) to the cartilage. The second type of Ab on the MSC surface acts against COL2. This initial connection of MSCs to COL2, as a main matrix part of hyaline cartilage that influences tissue integration^13^, can more confidently integrate MSCs into the cartilage matrix. In addition to preventing misattachment of C-TMN to other types of articular tissues (synovium, periosteom, and cartilage debris from enzymatic destruction of cartilage), tissue integration appears to be further enhanced. C-TMN-mediated binding of MSCs to cartilage can provide a 3D microenvironment for these MSCs and allow them to interact with matricellular proteins, growth factors, and other components of the cartilage extracellular matrix (ECM), all of which are essential for MSC engraftment and tissue integration^11^.

We hypothesize that targeted cell delivery can increase cellular involvement in the tissue regeneration process and dramatically increase the efficiency of cell therapy. By applying this strategy, we achieved integrated new cartilage, which is highly comparable to the original tissue in terms of function, radiological appurtenance, and OA scores. This therapeutic strategy can provide tremendous promise for the future of OA treatment.

## Results

### In vitro evaluation of mesenchymal stem cell (MSC) binding to a cell-tissue matchmaking nanoconstruct (C-TMN)

**Figure 1:**
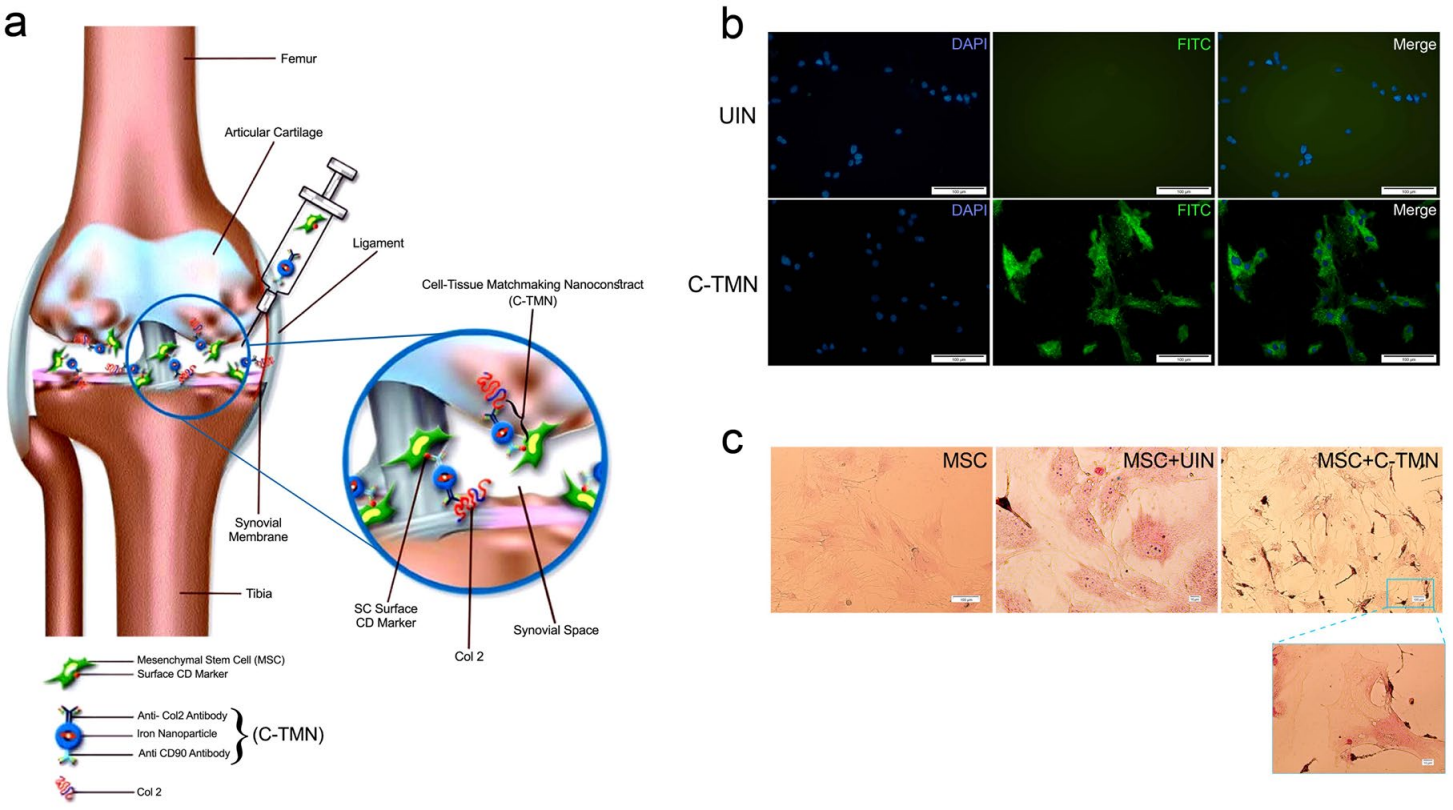
In vitro binding capacity/potential of cell-tissue matchmaking nanoconstructs (C-TMNs) to mesenchymal stem cells (MSCs). **(a)** General concept of cell-tissue matchmaking nanoconstructs (C-TMNs) and their theoretical role in mesenchymal stem cell (MSC)-cartilage binding. **(b)** Fluorescence microscopic images show the attachment of C-TMN but not unconjugated iron oxide nanoparticles (UINs) to MSCs (scale bars: 100 µM). MSC nuclei were stained with DAPI (blue), and FITC-labeled secondary antibody exhibited the attachment of C-TMN to the MSC surface (green). **(c)** Prussian blue staining of rat bone marrow MSCs (BMSCs) treated with UIN or C-TMN showed the binding of C-TMN but not UIN to the MSC surface. The image simultaneously shows endocytosis of UIN, but not C-TMN, by MSCs [scale bars: 100 µM (less magnification) and 10 µM (more magnification)].

Figure 1a shows the general schematic concept of C-TMN. Custom-built C-TMN was characterized, and its physicochemical properties were confirmed in vitro. Supporting Information Fig. S1 illustrates the scanning electron microscopy images of both C-TMN and unconjugated iron oxide NPs (Fe_3_O_4_; UIN). Dynamic light scattering (DLS) showed that C-TMN (123.1 ± 12.77 nm) was larger than UIN (61.81 ± 7.7 nm, Supporting Information Fig. S2).

Rat BMSCs were used for in vitro investigation of the specific binding ability of C-TMN to MSCs. The immunofluorescence assay (Fig. 1b) and Prussian blue iron staining (Fig. 1c) results showed that C-TMN could more efficiently bind to MSCs than UIN. In addition, Prussian blue staining results revealed that MSCs endocytosed UIN but not C-TMN.

### In vivo evaluation of cell-tissue matchmaking nanoconstruct (C-TMN)-cartilage binding

**Figure 2:**
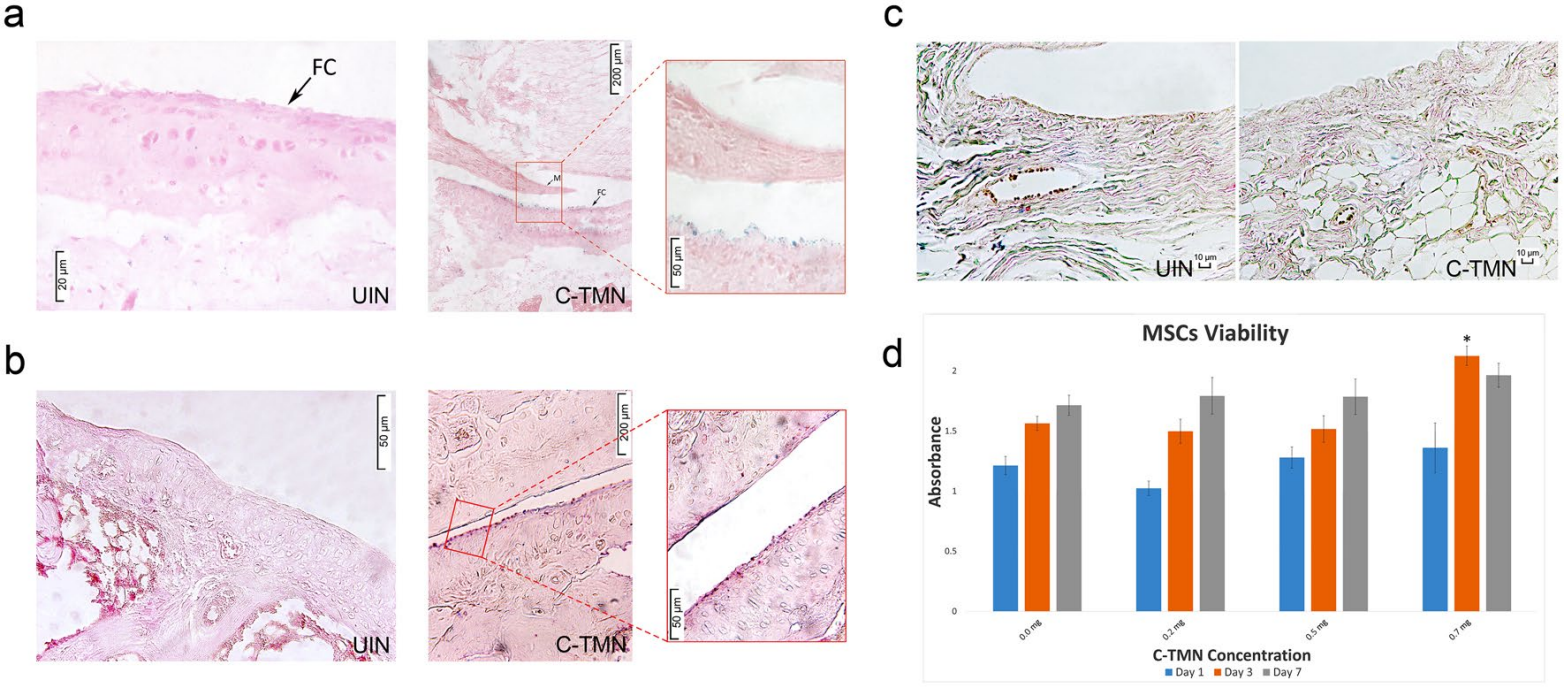
Cell-tissue matchmaking nanoconstruct (C-TMN) targets osteoarthritic and healthy articular cartilage (AC) in vivo. **(a)** Prussian blue staining shows specific binding of the cell-tissue matchmaking nanoconstruct (C-TMN) to osteoarthritic articular cartilage (OAC) (dark blue or purple spots on the cartilage surface) [scale bars: 20 µM (left), 200 µM (middle), 50 µM (right)]. **(b)** Specific binding of C-TMN to healthy AC is shown by Prussian blue staining [scale bars: 20 µM (left), 200 µM (middle), 50 µM (right)]. **(c)** Prussian blue staining demonstrates the absence of nonspecific binding of UIN or C-TMN to the osteoarthritic synovial membrane (scale bars: 10 µM). **(d)** The MTT assay results show that C-TMN does not affect the viability of mesenchymal stem cells (MSCs) at the desired dose (0.5 mg ml^−1^) (n = 3). *FC* fibrillated cartilage *Significant difference from the control at P ≤ 0.05.

We created a rat OA model using intra-articular (IA) injections of monosodium iodoacetate (MIA) to induce a partial cartilage defect. Because C-TMN was directed against the AC matrix, we induced OA at the level of a partial AC defect by selecting the appropriate MIA dosage (Supporting Information Fig. S3). To illustrate the binding ability of C-TMN to target tissue (rat AC cartilage), we injected C-TMN and UIN into the articular spaces of two rats with OA, and 24 h later, we used Prussian blue staining to track the C-TMN and UIN. Iron staining results demonstrated that C-TMN, but not UIN, was efficiently linked to both healthy (Fig. 2a) and osteoarthritic (Fig. 2b) cartilage since both tissues contained collagen II. This confirmed the specificity of C-TMN for AC. Prussian blue staining of the synovial membrane in the osteoarthritic rat knee joint showed a lack of nonspecific binding of UIN or C-TMN to the other acceptor surfaces in the joint (Fig. 2c). Conjugation of MSCs to C-TMN at the specified dose (0.5 mg ml^−1^) showed slight toxicity for MSCs, which was indicative of the safety of C-TMN (Fig. 2d).

### In vivo evaluation of the cell-tissue matchmaking nanoconstruct (C-TMN) to increase mesenchymal stem cell (MSC) therapy efficacy

The rat BMSCs had characteristics of MSCs, as shown by flow cytometry characterization of the surface epitopes and multipotentiality of these MSCs (Supporting Information Fig. S4). At seven weeks after the IA injection of MIA (four weeks after injection of different treatments), we tested the functional gain of C-TMN to engage the MSCs and target the AC. Assessments of gait analysis, histomorphometry and radiology, as well as molecular evaluation of cartilage tissue four weeks after treatments, revealed that targeted cell therapy with C-TMN increased AC targeting and improved the therapeutic outcome in the presence and absence of exogenous MSCs.

**Figure 3:**
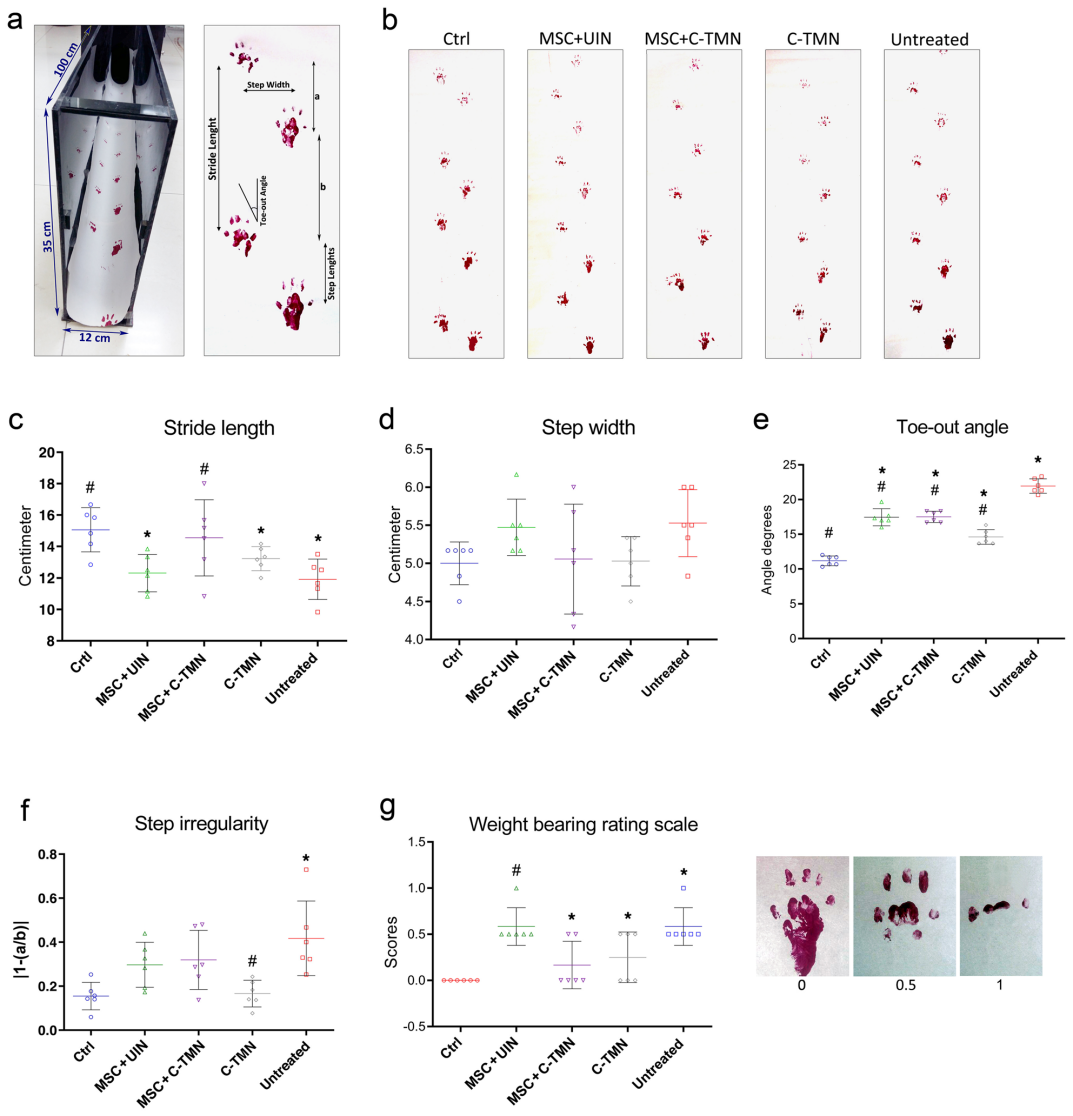
Footprint analysis of osteoarthritis (OA) model rats from spatial gate sequences in different groups and the effect of the mesenchymal stem cells (MSCs) and/or cell-tissue matchmaking nanoconstruct (C-TMN) on improvements of deficiencies in motor function. **(a)** Experimental setup of footprint recording (top left panel). Stride length, step width, toe-out angle, and step irregularity were measured as defined (right panel), and a visual rating scale was used to evaluate weight bearing (bottom left panel). **(b)** Representative footprints of five groups. Individual rats with painted hind paws were allowed to walk on the paper, and their footprints were evaluated. **(c)** Shorter stride length (the distance between two foot impacts of the same limb), **(d)** wider step width (transverse distance between the left and right paw), **(e)** larger toe-out angle, and **(f)** more step irregularity (two unequal consecutive step lengths) are representative of the severity of joint damage in that group in comparison with the control group. **(g)** Visual rating scale for weight bearing. Three modes of footprints were seen in this study that were scored as 0 (normal, equal weight on both hind paws), 0.5 (unequal toe pressure used to control the hind paws) and 1 (severely impaired weight bearing). The results are expressed as the mean ± SEM. *Significant difference compared with the control; ^#^Significant difference compared with the untreated group. P ≤ 0.05; (n = 6).

Gait analysis was carried out by reading the paw imprints before and after the desired treatments. Our analyzed data showed that C-TMN, but not UIN, with and without MSCs could reform most walking criteria, including stride length (the distance between two foot impacts of the same limb), step width (transverse distance between the left and right paw), toe-out angle, and step irregularity (two unequal consecutive step lengths) that had been damaged by OA (Fig. 3a–g).

**Figure 4:**
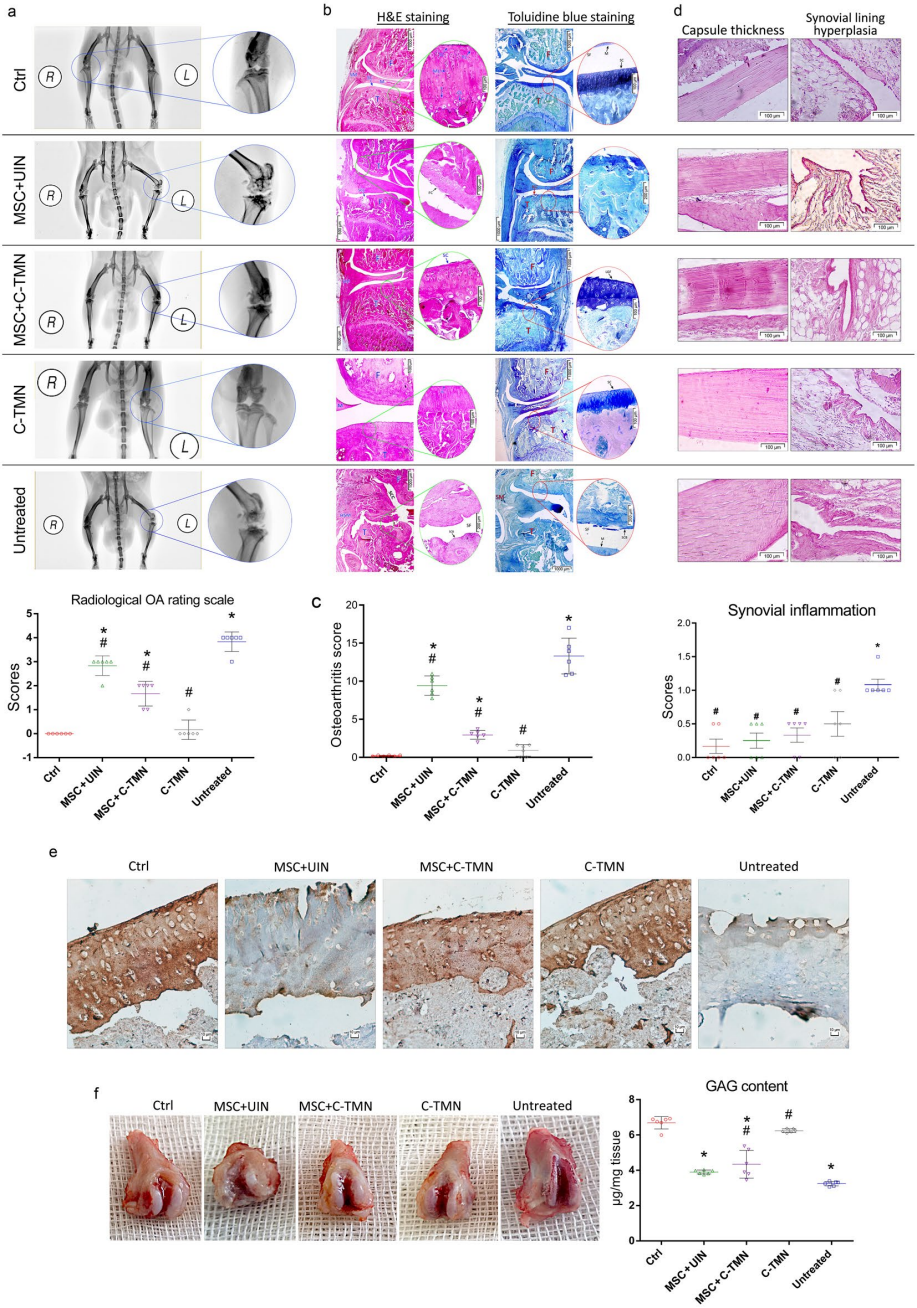
In vivo efficacy of cell-tissue matchmaking nanoconstructs (C-TMNs) in mesenchymal stem cell (MSC) targeting and osteoarthritic cartilage regeneration. **(a)** X-ray of the rat knee seven weeks after a monosodium iodoacetate (MIA) injection and representative semiquantitative chart for the radiological osteoarthritis rating scale. Posterior-anterior view of the whole body (left panel in each group) and a knee view at a higher magnification (right panel). All of the left knees in the different groups were induced with osteoarthritis (OA) followed by treatments. All of the right knees were the controls. *L* left, *R* right. The radiographs were scored based on a numerical rating scale^35^ (right). Cell-tissue matchmaking nanoconstructs (C-TMNs), but not exogenous mesenchymal stem cells (MSCs), had a significant impact on improving radiologic OA. *Significant difference compared with the control; ^#^Significant difference compared with the untreated group. P ≤ 0.01; (n = 6). **(b)** Histological analysis (hematoxylin and eosin [H&E] and toluidine blue staining) of the medial aspect of the rat femorotibial joints 28 days after MIA injection, which confirmed the therapeutic effect of C-TMN on the regeneration process of osteoarthritic articular cartilage (OAC). Changing the cartilage zonation (deletion of the middle layer), formation of a subchondral cyst, hypertrophic synovial membrane, fibrillated cartilage (H&E staining; capital letters) and proteoglycan loss (toluidine blue staining) were detected in the C-TMN-free group compared to the C-TMN-treated groups. The boxed areas in the left panels are magnified and displayed in the right circle panel [scale bars: 1000 µM (left panels), 100 µM (right panels)]. C-TMN with or without exogenous MSCs *F* femur, *T* tibia, *SM* synovial membrane, *M* meniscus, *SF* synovial fluid, *SC* smooth cartilage, *FC* fibrillated cartilage, *SCB* subchondral bone, *SCC* subchondral cyst, *HSM* hypertrophic synovial membrane, *SM* synovial membrane, *SL* superficial layer, *TL* transitional layer, *ML* middle layer, *T* tidemark, *CL* calcified layer. Magnification: left:  ×4, right:  ×40. Scale bar, left **(c)** semi-quantitative analysis of osteoarthritis (OA) severity. Manikin’s-derived histological scoring method for OACs. The OA score is the sum of the scores obtained from H&E and toluidine blue staining. *Significant difference with the control. ^#^Significant difference with the untreated group. P ≤ 0.05; (n = 6). **(d)** Histological analysis (H&E staining) of OA-induced synovitis (synovial lining hyperplasia (left, top row) and capsule thickness (left, bottom row) in rat articular cartilage (AC) seven weeks after MIA injection and representative semi-quantitative scoring (right, bottom row). The severity of synovitis was determined according to the suggested scoring system^14^. Note that MSCs are more effective in reducing inflammation than tissue repair. *Significant difference with the control. ^#^Significant difference with the untreated group. P ≤ 0.05; (n = 6) (scale bars: 100 µM). **(e)** Immunohistochemical (IHC) staining of repaired tissue for COL2 localization 28 days after C-TMN and/or MSC injection shows strong staining of cartilage in medial tibial plateaus treated with C-TMN. Note the weak expression of the COL2 protein in the C-TMN-free MSC therapy group compared with obvious expression in those groups with C-TMN-mediated targeted MSC therapy (scale bars: 10 µM). **(f)** Analysis of glycosaminoglycan (GAG) content in extracted rat AC determined by a Sulfated Glycosaminoglycan Assay kit (µg/mg cartilage) 28 days after the start of the treatments indicates higher levels of GAG reserve in C-TMN-treated cartilage. *Significant difference with the control. ^#^Significant difference with the untreated group. P ≤ 0.05; (n = 6).

Radiographic images of OA showed significant improvement in the group that received C-TMN compared with UIN and the untreated tissues (Fig. 4a). Histological analysis (hematoxylin and eosin [H&E], toluidine blue staining) showed major structural changes in the untreated and UIN-treated groups compared with the C-TMN-treated OAC group. Irregular surface discontinuity, disorientation of chondrocyte columns, mid- and deep loss of cells and matrix, osteophyte and cleft formation, and denudation of bone were the most frequent histologic changes observed (Fig. 4b,c). In addition, the decreased intensity of toluidine blue staining indicated a poor content of proteoglycans and glycosaminoglycan (GAG) in the UIN- and untreated AC compared with the C-TMN-treated AC (Fig. 4b). Measurement of inflammatory symptoms in the synovial membrane (synovitis), which was determined by synovial lining cell hyperplasia and joint capsule thickness^14^ revealed that synovitis was controlled in groups treated with MSCs or C-TMN compared to the untreated group, (Fig. 4d). A slight increase in synovitis score was seen in the C-TMN-treated group compared to the MSC-treated groups (MSC + UIN and MSC + C-TMN); however, this difference is not significant. The potential for C-TMN to enhance therapeutic benefits was further confirmed by immunohistochemical (IHC) analysis of the COL2 deposition pattern (Fig. 4e) and GAG content (Fig. 4f) in OA cartilage four weeks after treatment.

**Figure 5:**
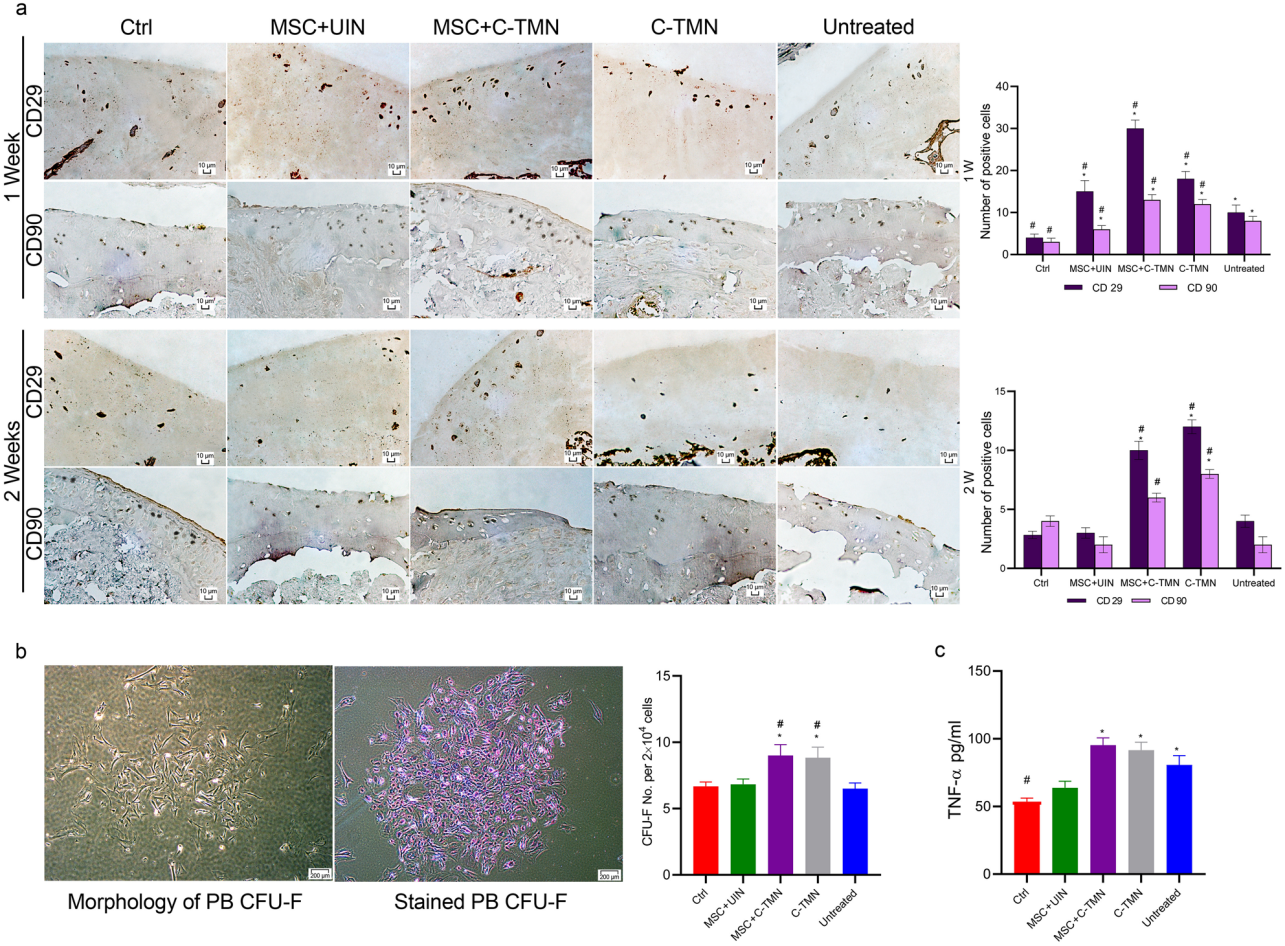
Identification of in vivo MSCs homing to osteoarthritic articular cartilage (OAC). **(a)** Immunohistochemistry staining of CD29 and CD90 in OACs at one and two weeks after treatment and related semi-quantification graphs of positively stained cells. The C-TMN and C-TMN + MSC groups had more CD29^+^ and CD90^+^ cells in the defect area at both time points (scale bars: 10 µM). **(b)** The number of colony forming unit fibroblasts (CFU-Fs) in rat peripheral blood one week after the injection and the related quantification graph (per 2 × 10^4^ plated cells) are shown. C-TMN increased the number of peripheral blood-derived CFU-Fs (n = 6 for each group, repeated in triplicate) (scale bars: 200 µM). **(c)** The TNF-alpha level in peripheral blood (pg ml^−1^) at one week post-treatment. The levels of TNF-alpha in the C-TMN and MSC + C-TMN groups were significantly increased compared to those in the MSC + UIN group. *TNF* tumor necrosis factor. The results are expressed as the mean ± SEM. *Significant difference compared with the control; ^#^Significant difference compared with the untreated group. P ≤ 0.05.

We also sought to address in vivo MSC homing to osteoarthritic AC. The AC tissue samples were subjected to immunohistochemical staining for CD29 and CD90 at one and two weeks after C-TMN and/or MSC injections. The results of numerous studies show that CD29 and CD90 are highly expressed by MSCs^15–17^. As shown in Fig. 5a, there were significant amounts of CD90^+^ and CD29^+^ cells in the C-TMN and MSC + C-TMN groups one week after the injection compared to the control, untreated and MSC + UIN groups. Although the numbers of MSC markers that expressed the cells dramatically decreased during the second week in all of the groups, their counts remained statistically higher in the C-TMN and MSC + C-TMN groups (Fig. 5b).

We assessed the concentration of TNF-alpha and the number of colony forming unit fibroblasts (CFU-Fs) in rat peripheral blood one week after C-TMN and/or MSC injection. The results indicated that treatment with C-TMN slightly increased TNF-alpha levels. MSCs secrete several cytokines, including TNF-alpha, to induce recruitment of endogenous MSCs^18^. The stimulatory effects of TNF-alpha on the migration of rat BM-MSCs were previously reported^19^. This result was accompanied by increased numbers of CFU-F (Fig. 5b,c). The CFU-F assay is indicative of the induction of systemic MSC mobilization. C-TMN-mediated entrapment of MSCs on the surface of osteoarthritic cartilage appeared to induce MSCs to secrete molecules involved in endogenous MSC recruitment, such as TNF-alpha, which led to increased numbers of CFU-Fs.

**Figure 6:**
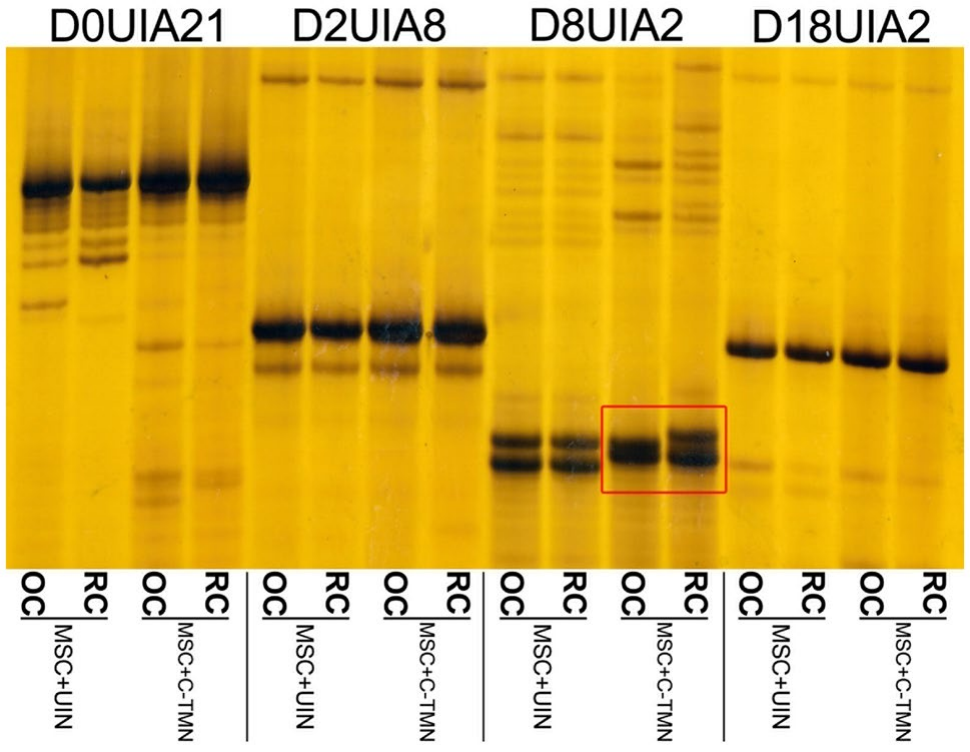
Short tandem repeat (STR) analysis used to identify the genetic source of the regenerated cartilage in groups that received exogenous mesenchymal stem cells (MSCs). Four rat DNA loci, D0UIA21, D2UIA8, D8UIA2, and D18UIA2, were used to check the genetic source of cartilage regeneration resulting tissues in the MSC + UIN and MSC + C-TMN groups. Different displays of D8UIA2 between regenerated cartilage (RC) and old cartilage (OC) in the MSC + C-TMN group (selected area in red) confirm the presence of exogenous MSCs in the composition of RC (n = 3 animals per group).

Finally, we performed short tandem repeat (STR) analysis to determine the parentage of the newly formed cartilage in groups that received exogenous MSCs: MSC + UIN and MSC + C-TMN (Fig. 6). For this aim, the origin of DNA in the chondrocytes of the regenerated cartilage was defined at four loci. For the group treated with MSC + UIN, we did not detect any DNA of the allogeneic MSCs for biopsies of four animals at the detection limit of the assay (1 in 1000 cells).

## Discussion

The therapeutic efficacy of C-TMN was greater in the absence of any exogenous MSCs, which indicated a high potential of C-TMN for in vivo precursor cell trapping. Thus far, multiple reports have discussed AC regeneration through targeted cell therapy using CTPs^9^ and bispecific antibodies (cell painting method)^7^. Both require cell surface modification, and their use in the clinic has been restricted. C-TMN does not have this limitation because its components (Ab and iron NPs) are safe to use in the clinic. As mentioned earlier, in vivo cell tracking for osteochondral tissues is difficult to perform with regular cell labeling methods, while C-TMN does not have this problem.

In this study, the presence of exogenous MSCs was associated with unexpected outcomes. When coinjected with C-TMN, MSCs decreased the regenerative efficiency of C-TMN; when injected alone, some evidence was found in favor of changing the AC zonation (deletion of the middle layer). MSCs simultaneously decreased inflammation in the articular space in the form of synovitis (hyperplasia of fibroblast-like synoviocytes), which was independent of C-TMN (both with and without C-TMN), implying the classic belief about the anti-inflammatory activity of MSCs, either exogenously injected or endogenously recruited into the articular space^20, 21^. Moreover, assessment of weight bearing, as a pain tolerance criterion, which was measured four weeks after treatment in the OA animals, showed that MSCs increased the ability to weight bear in these animals and could be the result of decreased inflammation. Therefore, it is believed that due to observed joint inflammation reduction in the presence of the exogenous MSCs, concomitant injection of C-TMN with MSCs may be more effective for both MSCs trapping and inflammation control.

On the other hand, the walking pattern in rats, which had changed because of OA progression, greatly improved only in the presence of C-TMN. In addition to the gait evaluation and histological tests, radiologic analysis also indicated progression of repair in the presence of C-TMN. This finding could provide evidence for improvements in both symptomatic and radiographic OA. Because a single cure for both features is rare, C-TMN could be considered an advanced, efficient approach for the clinical management of OA. We observed that even in the absence of exogenous MSCs, C-TMN injection led to promising therapeutic results. Although the present study did not provide any evidence for the involvement of MSCs in cartilage tissue repair, sequential assessments that confirmed the presence of MSCs/progenitor cells in OACs, as well as the higher numbers of PB CFU-F in animals treated with C-TMN that had improved OACs, demonstrated the possibility that exogenous or endogenous trapped MSCs contributed to cartilage repair. Thus, it is possible to treat OA in a less invasive manner through a one-step approach without the need for sampling or in vitro cell culturing.

At the nanoscale level, C-TMN can bind both endogenous recruited or exogenous MSCs to collagen II, which is a principal component of the microfibrillar network. C-TMN, a nanomatchmaker between MSCs and Coll II, may create a unique region around the trapped MSCs that can subsequently be part of the pericellular matrix (PCM) with a thickness of 2–4 µm that surrounds the final chondrocyte. The PCM is rich in proteoglycans, collagen (types II, IV and XI) and fibronectin^22^, and it modulates AC homeostasis^23^ by impacting signal transduction into chondrocytes^24, 25^ and maintaining chondrogenic differentiation^26^. Evidence supports the hypothesis that PCM can serve as a primary factor in both OA development and inhibition^27^. It has recently been reported that collagen II in the PCM can act as an extracellular signaling molecule to alter metabolic activity and inhibit chondrocyte hypertrophy^28^. In the current study, binding of MSCs to the AC matrix introduced collagen II into the MSC closed milieu at the beginning of the chondrocyte differentiation process. This resembles the conditions during which MSCs are treated with collagen and leads to clear promotion of MSC chondrogenic potential^29^. In addition to MSC trapping and localization at the surface of OA cartilage, C-TMN may promote MSC differentiation into chondrocytes by establishing the MSC-collagen interaction.

As the final step, STR analysis showed that, for the MSC + C-TMN group, three of the four rat DNA loci did not show any genetic polymorphism between host animal cells and cells obtained from regenerated cartilage, whereas one polymorphic marker (D8UIA2) exhibited the presence of exogenous cells in the newly formed cartilage. However, the identification of this informative marker was sufficient to confirm the presence of exogenous cell populations and their participation in the formation of new cartilage.

Here, we demonstrated that the attachment of C-TMN to the surface of osteoarthritic cartilage tissue, may also be a substrate for trapping endogenous progenitor cells in addition to exogenous MSCs. Our hypothesis was further supported when we observed that the peripheral blood samples collected from C-TMN treated animals had slightly increased concentration of TNF-α. According to the literature, TNF-α is not considered as an inflammatory marker of osteoarthritis in rat peripheral blood^30^. It has been reported that serum level of TNF-α in rat is associated with higher amount of endogenous recruited precursor cells^19^. However, since TNF-α is one of the important mediators of inflammatory response, more detailed pre-clinical studies in terms of C-TMN mediated inflammatory response and cytokine secretion profile are essential. Notably, we did not examine the exact pro-inflammatory cytokines profile neither before nor after injection of C-TMN; hence, more detailed assessments are required to ensure non-inflammatory consequences of this approach. In addition, further assessment is needed to evaluate the therapeutic potential of C-TMN in the absence of exogenous administered MSCs.

## Conclusion

In summary, our research group proposed a novel treatment approach for OA, during which fibrillated cartilage can be managed by a one-step approach, even in the absence of autologous biopsy or exogenous precursor cells. In this method, administration of C-TMN in the presence or absence of MSCs is directed in a simple classical way as a single IA injection that does not need to be repeated. Similar to other preliminary studies, this study should be confirmed by long-term studies because in OA, as a highly progressive disease, destruction may return despite early promising results.

## Methods

### In vitro cell-tissue matchmaking nanoconstruct-mesenchymal stem cell (MSC + C-TMN) binding test

BMSCs were isolated and characterized by flow cytometry for the surface epitopes and ability to differentiate into osteogenic and adipogenic lines (Supporting Information Fig. S4). To visualize the attachment of C-TMN to BMSCs, 5 × 10^4^ BMSCs were cultured in each well of six-well plates. We incubated BMSCs that were 70% confluent with C-TMN or unconjugated iron oxide nanoparticles (UIN) for 15 min at 37 °C. After washing, the MSCs were incubated with goat anti-rabbit FITC-labeled secondary antibody (ab6717, Abcam, USA) for 15 min at 37 °C. The efficacy of MSC + C-TMN binding was checked by an Olympus IX71 microscopic system.

We sought to determine whether C-TMN attached to the MSC surface or if it underwent endocytosis into MSCs. MSCs were incubated with C-TMN or UIN for 24 h at 37 °C for Prussian blue staining^31^. MSCs were fixed with 4% paraformaldehyde, stained using an iron staining kit (Sigma-Aldrich, USA), and visualized under a light microscope.

### In vitro assessment of cell-tissue matchmaking nanoconstruct (C-TMN) cytotoxicity

We examined the possible toxic effects of C-TMN on rat MSC viability and proliferation. Briefly, 5 × 10^4^ cells/ml were seeded in 96-well plates and incubated with 0.0, 0.2, 0.5, and 0.7 mg ml^−1^ C-TMN for 24 h at 37 °C. After the cells were washed, we assessed the proliferation capacity of the MSCs with the 3-(4,5-dimethylthiazol-2-yl)-2,5-diphenyltetrazolium bromide (MTT) assay. We added MTT solution (500 µg ml^−1^) to each well and incubated the plates for 3 h. Formazan crystals were dissolved in DMSO, and the intensity of the MTT product was measured at 540 nm using a microplate reader.

### Induction of osteoarthritis (OA) in the rats

All experiments on male Wistar rats were conducted according to the National Institute of Health Guide for the Care and Use of Laboratory Animals and were approved by the Ethics Committee for Animal Experimentation of Royan Institute (code: IR.ACECR.ROYAN.REC.1394.159). This study is also reported in accordance with ARRIVE guidelines.

Male rats that were greater than 8 weeks old and weighed 200–250 g were used to create an animal OA model. OA was induced by a single IA injection of monosodium iodoacetate (MIA) at doses of 1, 2 or 3 mg per 150 g body weight^32^. Briefly, MIA (Sigma-Aldrich, USA) was dissolved in PBS/physiological saline and injected with a 26-gauge 0.5-inch needle at a volume of 50 μl into the left knee joints. The desired level of damage (partial cartilage defect) was observed only at the 2 mg dose. The 1 mg dose induced slight surface fibrillation, and the 3 mg dose induced full-thickness cartilage loosening. Three weeks after the MIA injection (desired duration for induction of OA), we conducted gait analysis, imaging, and histological tests to confirm the presence of OA in the rats.

### In vivo assessment of cell-tissue matchmaking nanoconstruct (C-TMN) attachment to cartilage

To evaluate binding efficacy, C-TMN (0.5 mg ml^−1^) or UIN (0.5 mg ml^−1^) was injected into the articular spaces of both healthy and OA rats. After 24 h, the animals were sacrificed. The hind limbs were disarticulated at the knee joint, and the knees were fixed in 10% neutral-buffered formalin for 48 h at 4 °C and decalcified for eight days with 5% nitric acid. The samples were then dehydrated, embedded in paraffin, and cut into 6-μm-thick frontal sections^31^. Tissue samples were stained separately with Prussian blue to determine if the iron nanoconstruct attached to the cartilage surface or synovial membrane.

### Measuring the in vivo efficacy of cell-tissue matchmaking nanoconstructs (C-TMNs) on cartilage repair

The OA model rats were randomly divided into four experimental groups (6 animals per group): MSC + C-TMN, C-TMN, MSC + UIN, and untreated three weeks after the MIA injection. In each experimental group, the right knees were kept intact and considered to be the Ctrl in all figures and histograms. Three weeks after the MIA injection, all rats in the MSC + C-TMN group received IA injections of 50 µl of C-TMN (0.5 mg ml^−1^) into their left knee joints using a 26-gauge 0.5-inch needle. After 20 min, they received IA injections of 1 × 10^6^ allogeneic BMSCs from 6–8-week-old male rats. The UIN + MSC group animals received IA injections of 50 µl of UIN (0.5 mg ml^−1^) into the left knee joints three weeks post-MIA injection, followed 20 min later by an IA injection of 1 × 10^6^ allogeneic BMSCs suspended in 50 µl PBS (from 6 to 8-week-old male rats). The OA animals in the C-TMN group received IA injections of 50 µl of C-TMN (0.5 mg ml^−1^) into the left knee joints 20 min later by injections of 50 µl PBS. In the untreated group, the left MIA-injected knees received twice 50 µl PBS at the same time as C-TMN or UIN and MSC injection. After four weeks, cartilage health and joint function were examined by gait analysis, radiologic imaging, and histologic and immunohistologic analyses in all of the groups.

### Gait analysis

To perform the final analysis four weeks after treatment, a walking track as a motor behavior test was carried out using the geometric characteristics of a paw print during locomotion (spatial gait data)^33^. Briefly, the hind paws of the rats were painted, and the animals were placed at the entrance of the 120 mm long dark tunnel covered with paper. The animals were allowed to walk along the tunnel, and their footprints on the paper were evaluated. At least four complete gait cycles were recorded on each paper in a single trip, and we analyzed at least three artifact-free gait cycles for each animal. The spatial gate variables included the toe-out angle (the angle between orientation of the progression and a reference line in through the foot heel and the third digit), stride length (the distance between two paw prints of the same limb), step width, and step irregularity (two unequal consecutive step lengths). Figure 3a represents the experimental setup of footprint recording and scoring parameters. The mean of three complete gait cycles was analyzed in each animal for all gate analysis parameters. Visual scoring of weight bearing was performed by using a paw pressure rating scale^34^. Three modes of footprints were seen in this study and scored as 0 (normal, equal weight on both hind paws), 0.5 (unequal toe pressure used to control the hind paws) and 1 (severely impaired weight bearing). The footprint papers were scanned, and we measured the spatial variables by a blind pathologist.

### X-ray and histopathological analysis

The radiology procedure was carried out using a micro X-ray while the rats were placed under general anesthesia to evaluate the severity of OA before they were euthanized. Radiographs were obtained in the dorsolateral and anteroposterior positions, and the resultant images were scored based on a numerical rating scale^35^. Briefly, any alteration in joint space, osteophyte formation and subchondral sclerosis were evaluated and each radiograph was assigned a grade from 0 to 4; 0 = no signs of OA related tissue change; 1 = minimal osteophytes; 2 = decreased joint space and distinct osteophytes; 3 = narrow joint space and moderate osteophyte formation and 4 = subchondral sclerosis and significant reduction in joint space.

Subsequently, six animals per group were sacrificed, and all of the hind limbs were disarticulated at the knee joint. Paraffin blocks were prepared and cut into 6-μm-thick frontal sections to be used for H&E staining. Toluidine blue (a specific staining for cartilage) staining and immunohistochemistry analysis were performed to visualize the collagen II and aggrecan contents of AC in the different groups. Semi-quantitative histopathologic scoring was based on a histological scoring system by Mankin et al. (1971), where the surface smoothness or fibrillation, population and orientation of the chondrocytes, chondrocyte death or hypertrophy, and loss of matrix were considered to be the main histological features for numeric grading. Briefly, normal cartilage tissue received score 0, cartilage with rough surface received score 1, cartilage surface interruption received score 2, and score 3, 4 and 5 were considered for cleft formation, erosion and denudation in cartilage, respectively.

Toluidine blue staining was performed to evaluate the proteoglycan content of the cartilage^36^ and based on color intensity each slide were scored as 0 = normal, 1 = slight reduction, 2 = moderate reduction, 3 = sever reduction and 4 = no dye noted^36^. The sum of the scores obtained from both H&E and toluidine blue staining was considered to be the final OA score.

Inflammation in the synovial membrane (synovitis) was determined by synovial lining cell count and joint capsule thickness and was quantified using numerical scoring method by Mapp et al.^14^. In this method, the ranging from 0 to 3 was applied for scoring of synovial cellularity, where the 1–2 cell thick lining cell layer receives score 0, 3–5 cell thick lining cell layer receives score 1, 6–8 cell thick lining cell layer receives score 2 and > 9 cell thick lining cell layer receives score 3.

All scoring was conducted by a pathologist who was unaware of the treatment assignments. We used the mean value of two sections for histological staining data (H&E and TB) for each animal.

The GAG content of AC was determined with a Sulfated Glycosaminoglycan Assay kit (Blyscan™, Biocolor, UK) according to the manufacturer’s protocol. Measurement of spectrophotometer absorbances was performed at 656 nm, and the values were expressed as micrograms of GAG per milligram of cartilage wet weight.

### Immunohistochemical staining

We evaluated the expression levels of CD29 and CD90 (as MSC surface markers; one and two weeks after treatments) and COL2 (as a chondrogenic marker; four weeks after treatments) by immunohistochemical analysis. The hind limbs of the rats were disarticulated at the knee joints. Next, the knees were fixed in 10% neutral-buffered formalin for 48 h at 4 °C and decalcified for eight days in 5% nitric acid. The samples were then dehydrated, embedded in paraffin, and cut into 6-μm-thick frontal sections. All specimens (n = 6 per group) were immersed in citrate buffer (pH 6) as an antigen retrieval solution at 65 °C. Nonspecific staining was blocked by 30 min incubation of the sections with 10% bovine serum albumin (BSA) and subsequently incubated overnight at 4 °C with primary antibodies against rat CD29 (555005, BD Bioscience, USA) and CD90 (ab225, Abcam, USA) as well as collagen II (250484, Abbiotec, USA). Horseradish peroxidase-conjugated secondary antibodies (Invitrogen, A-11010, USA) were dropped onto slides (1 h, room temperature). The immunoreaction was visualized by the addition of a 0.02% hydrogen peroxide solution (DAB Substrate Chromogen System; X3468, Dako, USA). The images were taken under a light microscope and evaluated using Image-Pro Plus 6.0 software.

### Colony forming unit-fibroblasts (CFU-F) assay

One week after the C-TMN and/or MSC injections, we collected peripheral blood from the control and experimental group rats (6 samples per group) following intraperitoneal (IP) administration of ketamine (40–100 mg kg^−1^) and xylazine (5–13 mg kg^−1^ body weight). For the colony-forming unit fibroblast (CFU-F) assay, rat peripheral blood mononuclear cells (6 samples per group) were plated in six-well plates at densities of 2 × 10^4^ cells per well. The adherent colonies (> 50 cells) derived from the CFU-Fs were stained with crystal violet and counted after 10 days. Assessment of each group was performed in triplicate.

### Measurement of TNF-alpha concentration

One week after C-TMN and/or MSC injections, TNF-alpha levels were measured in blood collected from the intraperitoneal vein of rats. For this aim, the blood samples were centrifuged at 630 × *g* for 15 min at 4 °C and preserved at −80 °C. The concentrations of TNF-alpha were measured by a quantitative ELISA kit (Demeditec, DE4641, Germany) and reported as pg/ml blood serum.

### Short tandem repeat (STR) analysis

We performed STR analysis in the groups with exogenous MSCs (UIN + MSC and MSC + C-TMN). Genomic DNA was extracted from two sources: the superficial cartilage part of the left knee joints (regenerated cartilage of the OA joint treated with MSCs) and the superficial cartilage part of the right knee joints (untreated old cartilage) after the decalcification process using a DNeasy Blood & Tissue Mini Kit (Qiagen, Germany). Four rat DNA loci (D0UIA21, D2UIA8, D8UIA2, and D18UIA2) were amplified^37^, and specific alleles for the MSC donor and host were determined. The length of the short tandem repeat (STR) amplicons was compared in both sources of biopsies (repaired cartilage from the left knee joint and old cartilage from the right knee joint) to identify the parentage of the cells in the repaired tissues.

### Statistical analysis

Statistical analyses were performed using GraphPad Prism software, version 8 (GraphPad Software, San Diego, CA). The results from the animal studies are presented as the mean ± standard error (SE). Data normality was assessed by the Kolmogorov–Smirnov test. Comparisons between more than two groups were carried out using one-way analysis of variance (ANOVA). Differences between each pair of groups were evaluated using the Tukey test. Statistical significance was defined at P < 0.05.

## Supplementary Information

Supplementary Information.

## Abbreviations

MSCs: Mesenchymal stem cells
OA: Osteoarthritis
C-TMN: Cell-tissue matchmaking nanoconstruct
NP: Nanoparticle
AC: Articular cartilage
MIA: Monosodium iodoacetate
IA: Intra-articular
ACI: Autologous chondrocyte implantation
BM: Bone marrow
OAC: Osteoarthritic AC
Ab: Antibody
CTPs: Cell targeting peptides
COL2: Collagen type II
ECM: Extracellular matrix
DLS: Dynamic light scattering
UIN: Unconjugated iron oxide
GAG: Glycosaminoglycan
PCM: Pericellular matrix
STR: Short tandem repeat

## References

[1] Zhang Y, Jordan JM (2010). Epidemiology of osteoarthritis. Clin. Geriatr. Med..

[2] Diekman BO, Guilak F (2013). Stem cell-based therapies for osteoarthritis: Challenges and opportunities. Curr. Opin. Rheumatol..

[3] Weissman IL (2000). Translating stem and progenitor cell biology to the clinic: Barriers and opportunities. Science.

[4] Curley GF (2012). Mesenchymal stem cells enhance recovery and repair following ventilator-induced lung injury in the rat. Thorax.

[5] Satue M, Schuler C, Ginner N, Erben RG (2019). Intra-articularly injected mesenchymal stem cells promote cartilage regeneration, but do not permanently engraft in distant organs. Sci. Rep..

[6] Nasiri N, Hosseini S, Alini M, Khademhosseini A, Baghaban Eslaminejad M (2019). Targeted cell delivery for articular cartilage regeneration and osteoarthritis treatment. Drug Discov Today.

[7] Dennis JE, Cohen N, Goldberg VM, Caplan AI (2004). Targeted delivery of progenitor cells for cartilage repair. J. Orthop. Res..

[8] Pi Y (2011). Targeted delivery of non-viral vectors to cartilage in vivo using a chondrocyte-homing peptide identified by phage display. Biomaterials.

[9] Hu HY (2015). DOTAM derivatives as active cartilage-targeting drug carriers for the treatment of osteoarthritis. Bioconjug. Chem..

[10] Ansboro S, Greiser U, Barry F, Murphy M (2012). Strategies for improved targeting of therapeutic cells: Implications for tissue repair. Eur. Cell Mater..

[11] Zhao J (2014). Stem cell-mediated delivery of SPIO-loaded gold nanoparticles for the theranosis of liver injury and hepatocellular carcinoma. Nanotechnology.

[12] Cheng K (2014). Magnetic antibody-linked nanomatchmakers for therapeutic cell targeting. Nat. Commun..

[13] Khan IM, Gilbert SJ, Singhrao SK, Duance VC, Archer CW (2008). Cartilage integration: Evaluation of the reasons for failure of integration during cartilage repair. A review. Eur. Cell Mater..

[14] Mapp PI (2013). Differences in structural and pain phenotypes in the sodium monoiodoacetate and meniscal transection models of osteoarthritis. Osteoarthritis Cartil..

[15] Deng J (2013). A silk fibroin/chitosan scaffold in combination with bone marrow-derived mesenchymal stem cells to repair cartilage defects in the rabbit knee. J. Mater. Sci. Mater. Med..

[16] Tan SL, Ahmad TS, Selvaratnam L, Kamarul T (2013). Isolation, characterization and the multi-lineage differentiation potential of rabbit bone marrow-derived mesenchymal stem cells. J. Anat..

[17] Yang J (2017). Regulation of the secretion of immunoregulatory factors of mesenchymal stem cells (MSCs) by collagen-based scaffolds during chondrogenesis. Mater. Sci. Eng. C Mater. Biol. Appl..

[18] Zhang S (2020). Articular cartilage regeneration: The role of endogenous mesenchymal stem/progenitor cell recruitment and migration. Semin. Arthritis Rheum..

[19] Naaldijk Y (2015). Migrational changes of mesenchymal stem cells in response to cytokines, growth factors, hypoxia, and aging. Exp. Cell Res..

[20] Khubutiya MS, Vagabov AV, Temnov AA, Sklifas AN (2014). Paracrine mechanisms of proliferative, anti-apoptotic and anti-inflammatory effects of mesenchymal stromal cells in models of acute organ injury. Cytotherapy.

[21] Sicco CL (2017). Mesenchymal stem cell-derived extracellular vesicles as mediators of anti-inflammatory effects: Endorsement of macrophage polarization. Stem Cells Transl. Med..

[22] Miosge N (1994). Light and electron microscopical immunohistochemical localization of the small proteoglycan core proteins decorin and biglycan in human knee joint cartilage. Histochem. J..

[23] Chen C, Tambe DT, Deng L, Yang L (2013). Biomechanical properties and mechanobiology of the articular chondrocyte. Am. J. Physiol. Cell Physiol..

[24] Halloran JP (2012). Multiscale mechanics of articular cartilage: Potentials and challenges of coupling musculoskeletal, joint, and microscale computational models. Ann. Biomed. Eng..

[25] Khoshgoftar M, Torzilli PA, Maher SA (2018). Influence of the pericellular and extracellular matrix structural properties on chondrocyte mechanics. J. Orthop. Res..

[26] Peters HC (2011). The protective role of the pericellular matrix in chondrocyte apoptosis. Tissue Eng. Part A.

[27] Loeser RF, Collins JA, Diekman BO (2016). Ageing and the pathogenesis of osteoarthritis. Nat. Rev. Rheumatol..

[28] Lian C (2019). Collagen type II suppresses articular chondrocyte hypertrophy and osteoarthritis progression by promoting integrin beta1-SMAD1 interaction. Bone Res..

[29] Tamaddon M (2017). Monomeric, porous type II collagen scaffolds promote chondrogenic differentiation of human bone marrow mesenchymal stem cells in vitro. Sci. Rep..

[30] Collins KH (2015). Relationship between inflammation, the gut microbiota, and metabolic osteoarthritis development: Studies in a rat model. Osteoarthritis Cartil..

[31] Cheng K (2010). Magnetic targeting enhances engraftment and functional benefit of iron-labeled cardiosphere-derived cells in myocardial infarction. Circ. Res..

[32] Mohan G (2011). Application of in vivo micro-computed tomography in the temporal characterisation of subchondral bone architecture in a rat model of low-dose monosodium iodoacetate-induced osteoarthritis. Arthritis Res. Ther..

[33] Beckett J (2012). Excessive running induces cartilage degeneration in knee joints and alters gait of rats. J. Orthop. Res..

[34] Angeby Moller K, Kinert S, Storkson R, Berge OG (2012). Gait analysis in rats with single joint inflammation: influence of experimental factors. PLoS ONE.

[35] Morais SV (2016). Osteoarthritis model induced by intra-articular monosodium iodoacetate in rats knee. Acta Cir. Bras..

[36] Ostergaard K, Andersen CB, Petersen J, Bendtzen K, Salter DM (1999). Validity of histopathological grading of articular cartilage from osteoarthritic knee joints. Ann. Rheum. Dis..

[37] Walder RY (1998). Short tandem repeat polymorphic markers for the rat genome from marker-selected libraries. Mamm. Genome.

