# Supplementary material for: Targeted mesenchymal stem cell therapy equipped with a cell-tissue nanomatchmaker attenuates osteoarthritis progression
Source: Sci Rep. 2022 Mar 7;12:4015. doi: 10.1038/s41598-022-07969-9 (PMC8901617; doi:10.1038/s41598-022-07969-9)
Supplement: Supplementary file 1 — Supplementary Information. [file 41598_2022_7969_MOESM1_ESM.docx]

Supplementary Information

| **Targeted Mesenchymal Stem Cell Therapy Equipped with Cell-tissue Nanomatchmaker Attenuates Osteoarthritis Progression** |
| --- |
| Nahid Nasiri^1, 2^, Samaneh Hosseini^1,3^, Fakhreddin Reihani-Sabet^4^, Mohamadreza Baghaban Eslaminejad^1^*(**🖂**)  *^1^Department of Stem Cells and Developmental Biology, Cell Science Research Center, Royan Institute for Stem Cell Biology and Technology, ACECR, Tehran, Iran*  *^2^Department of Embryology, Reproductive Biomedicine Research Center, Royan Institute for Reproductive Biomedicine, ACECR, Tehran, Iran*  *^3^Department of Cell engineering, Cell Science Research Center, Royan Institute for Stem Cell Biology and Technology, ACECR, Tehran, Iran*  *^4^Department of Genetics, Reproductive Biomedicine Research Center, Royan Institute for Reproductive Biomedicine, ACECR, Tehran, Iran* |

Material

We ordered a nanotechnology company (NVIGEN, Inc. CA, USA) to synthesize Cell-tissue matchmaking nanoconstruct (C-TMN). The custom built C-TMN was structurally composed of two types of antibodies: rabbit anti-CD90 (Abcam, USA) and rabbit anti-collagen II (Abcam, USA); both were covalently conjugated to a central iron nanoparticle (NP). The product attached information included C-TMN size (115.8 nm) and spectrophotometric measurement of protein (antibody [Ab]) concentration in pre-conjugation media (1.7984) and post-conjugation (0.1772) media at 280 nm (A280).

MSC isolation and characterization: BMSCs were isolated from 6–8 weeks old male Wistar rats by femur and tibiae bone flushing and gradient centrifugation (1200 rpm, 5 min) (Takahashi, Li et al., 2006). The rat BMSCs were then cultured as monolayer in DMEM culture medium (Gibco) supplemented with 1% penicillin/streptomycin and 15% heat-inactivated FBS. The third passage BMSCs were characterized using assessment of their adipogenic (oil red O staining) and osteogenic (alizarin red S staining) potential, immunophenotyping by flow cytometery and confirmation of fibroblast-like morphology.


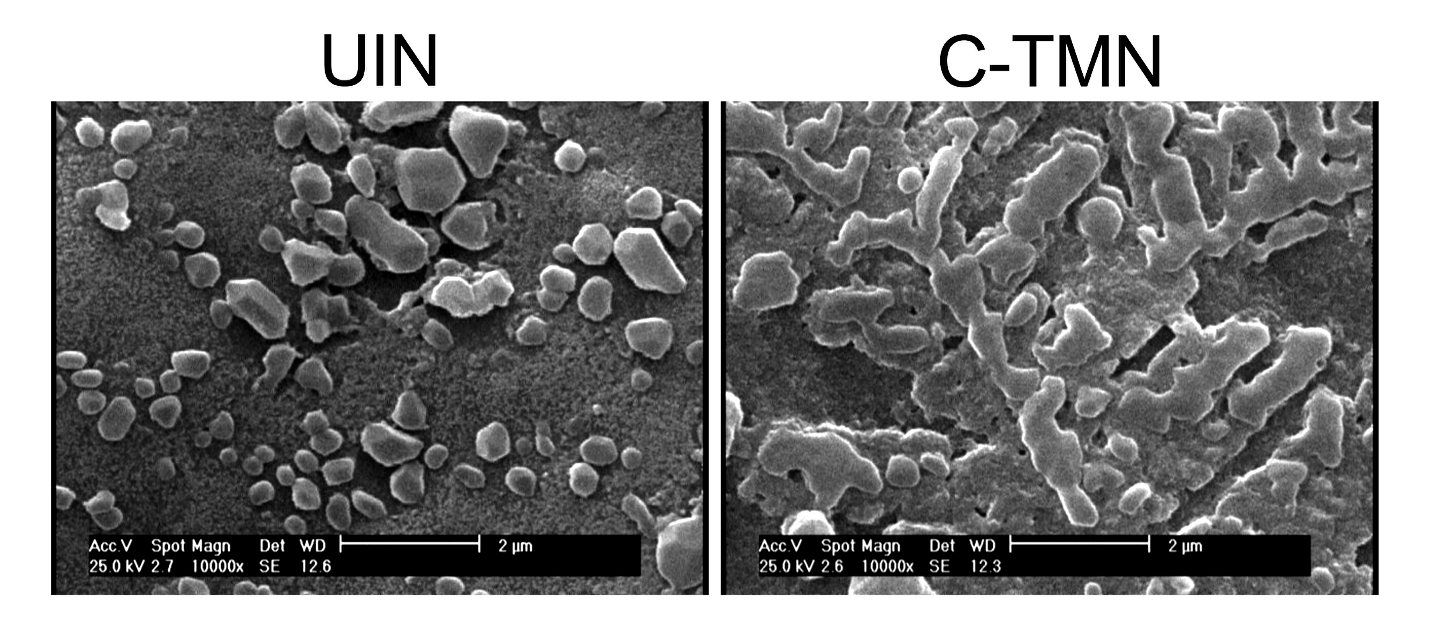


**Figure S1**. Scanning electron microscopy (SEM) images of unconjugated iron oxide nanoparticle (UIN) and cell-tissue matchmaking nanoconstruct (C-TMN). The micrographs confirm modification of the nanoparticle (NP) surface following conjugation with antibody (Ab).


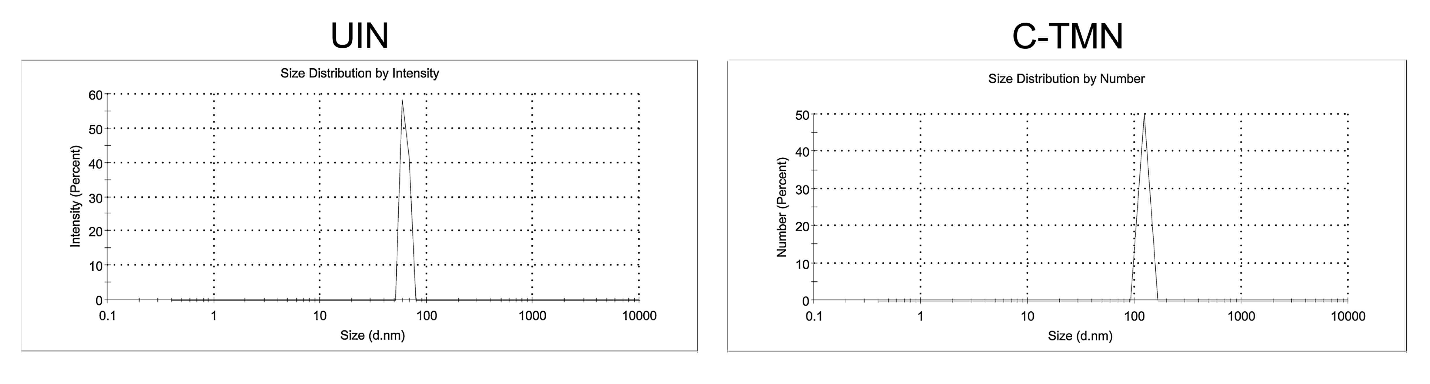


**Figure S2.** Dynamic light scattering (DLS) images show the average size distributions of the unconjugated iron oxide nanoparticle (UIN; 61.81±7.7 nm) and cell-tissue matchmaking nanoconstruct (C-TMN; 123.1±12.77 nm).


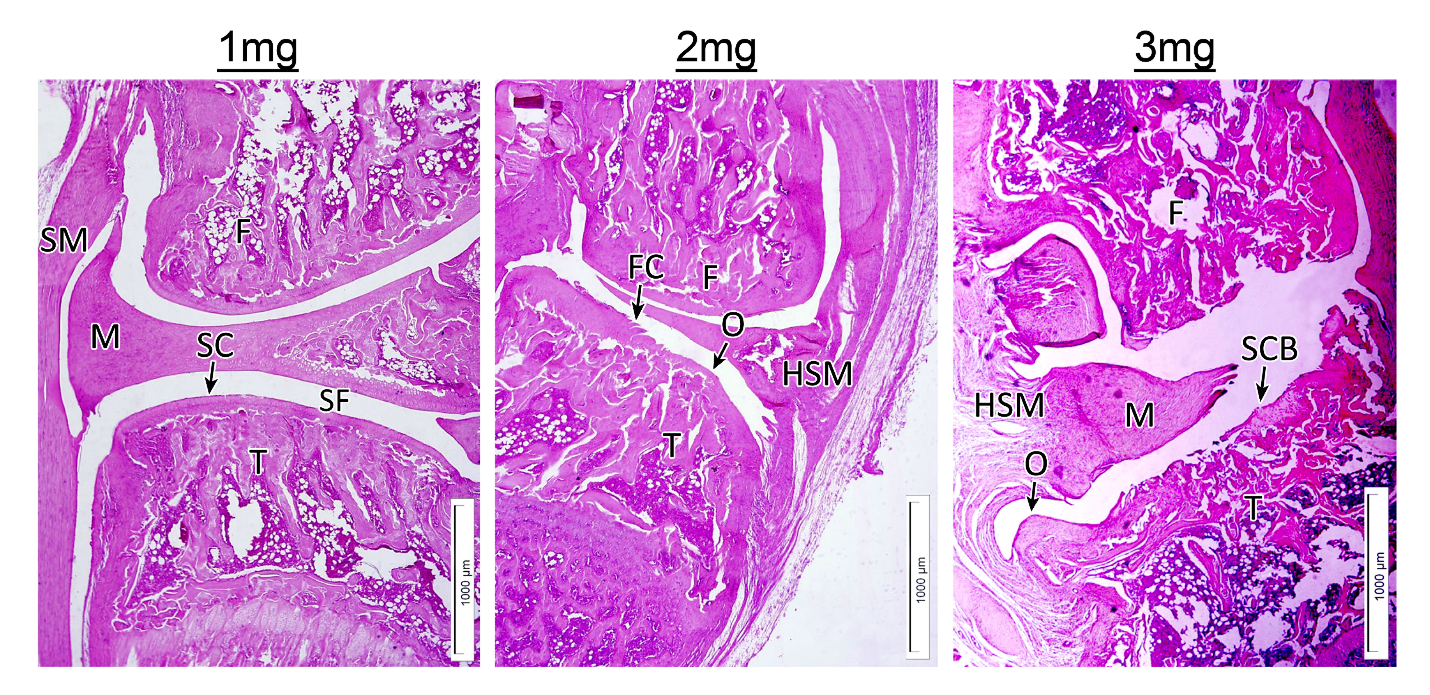


**Figure S3.** Selection of the proper dose of monosodium iodoacetate (MIA) to create a partial cartilage defect in the adult rat knee joint followed by osteoarthritis (OA) development. Three doses (1, 2, and 3 mg) of monosodium iodoacetate (MIA) per 50µl of PBS- were studied and the resultant cartilage defects were assessed. Hematoxylin and eosin (H&E) staining of the medial aspect of the rats’ femorotibial joints shows that the partial cartilage defects only occurred at the 2 mg of MIA dose in 50 µl PBS-. Doses 1 and 3 did not cause cartilage defects or induce full thickness cartilage loose, respectively. At the 2 mg dose, OA progression was confirmed after detection of synovial hypertrophy, osteophyte formation, and cartilage fibrillation (scale bars: 1000 µM). F: Femur, T: Tibia, HSM: Hypertrophic synovial membrane, M: Meniscus, SCB: Subchondral bone, O: Osteophyte, SF: Synovial fluid, FC: Fibrillated cartilage, SC: Smooth cartilage, SM: Synovial membrane. Scale bar: 100 µm


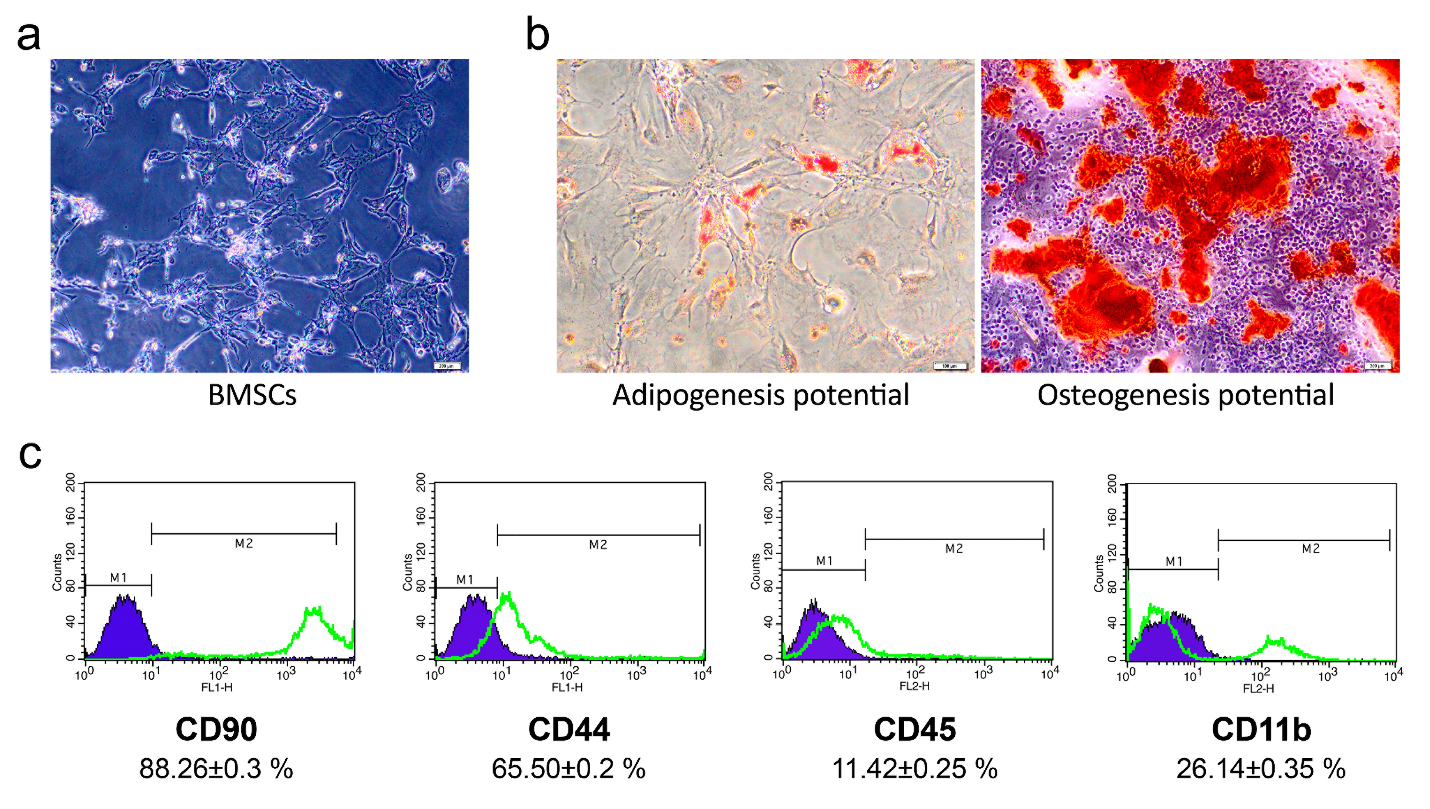


**Figure S4.** Characterization of rat bone marrow mesenchymal stem cells (BMSCs). (a) Morphology of the passage-3 rat bone marrow mesenchymal stem cells (BMSCs) (scale bars: 200 µM). (b) Confirmation of adipogenic and osteogenic potential of rat BMSCs by cytoplasmic lipid droplets with oil red O and appearance of mineralized nodular structures by alizarin red S staining, respectively (scale bars: 100 µM (left), 200 µM (right)). (c) Analysis of passage-3 rat BMSC immunophenotype by flow cytometry for CD90, CD44, CD45, and CD11b expressions.
